# Retrosplenial cortex 5-HT2A receptors critically contribute to recognition memory processing

**DOI:** 10.3389/fncel.2025.1711777

**Published:** 2025-11-18

**Authors:** Beatriz Agustina Ortega, Noelia V. Weisstaub, Cynthia Katche

**Affiliations:** 1 Laboratorio de Memoria, Instituto de Biología Celular y Neurociencia “Prof. E. DeRobertis” (IBCN), Facultad de Medicina, CONICET-UBA, Buenos Aires, Argentina; 2 Instituto Tecnológico de Buenos Aires (ITBA), Buenos Aires, Argentina; 3 Instituto de Neurociencia Cognitiva y Traslacional (INCYT), CONICET- Fundación INECO, Universidad Favaloro, Buenos Aires, Argentina

**Keywords:** retrosplenial cortex, recognition memory, serotonin receptor 2A, memory consolidation, memory retrieval

## Abstract

The anterior retrosplenial cortex (aRSC) functions as a hub that integrates multimodal sensory inputs into associative recognition memories. Although the aRSC receives dense serotonergic projections from the raphe nuclei, the role of serotonin in its function remains poorly understood. Among serotonergic receptors, 5-HT2A receptors (5-HT2ARs) are highly expressed in cortical regions, including the aRSC, and have been implicated in the modulation of cognitive processes. Based on our previous work demonstrating the involvement of the aRSC in recognition memory, here we investigated the contribution of 5-HT2ARs (memory) during different phases of the object recognition (OR) task in rats. We found that selective blockade of 5-HT2ARs in the aRSC differentially affected acquisition, consolidation, and retrieval. These findings identify 5-HT2ARs in the aRSC as critical modulators of recognition memory processing and suggest that their dysregulation could contribute to cognitive impairments observed in conditions such as Alzheimer’s disease.

## Introduction

The retrosplenial cortex (RSC) has traditionally been linked to the processing of various variables related to actions, visual stimuli, spatial location, and orientation (Cho and Sharp, 2001). While it is primarily known for its role in navigation through such spatial and directional coding, its functions extend well beyond this domain. A growing body of research highlights the RSC’s involvement in memory processes, particularly in the encoding and retrieval of spatial, sequence-based, or “episodic,” memories (Vann et al., 2009; Miller et al., 2014; Koay et al., 2022; Stacho and Manahan-Vaughan, 2022; Alexander et al., 2023; Subramanian and Smith, 2024). The RSC represents the most caudal region within the cortical band surrounding the corpus callosum, commonly known as the cingulate cortex. In primates, this cingulate cortex is typically divided into anterior and posterior sections. The RSC corresponds to the most caudoventral portion of the posterior cingulate cortex. In contrast, in rodents, the RSC encompasses the entire posterior cingulate region (Vogt and Peters, 1981; Vann et al., 2009). The RSC is sensitive to changes that occur in mild cognitive impairment (Trask and Fournier, 2022) and in the early stages of Alzheimer’s disease (AD) (Pengas et al., 2010; Aggleton, 2014; Tu et al., 2015), making understanding its function crucial. Particularly, the anterior RSC (aRSC) is a hub that integrates multimodal sensory information into cohesive associative recognition memories (Aggleton, 2014; Trask and Fournier, 2022).

Serotonin (5-HT) plays a fundamental role in cognition, emotional regulation and motivated behaviors (Barrett et al., 2017) and changes in serotonergic system are associated with mild cognitive impairment (MCI) and AD (Palmer et al., 1988; Hirao et al., 2015). Interestingly, the aRSC receives significant serotonergic projections from the raphe nucleus, particularly at its rostral pole (Lidov et al., 1980; Awasthi et al., 2021). In humans, changes in resting state connectivity associated with manipulations of serotonin levels, between aRSC and prefrontal regions and aRSC and Parahipocampal regions have been observed supporting a critical role for the serotonergic system in the modulation of aRSC mediated functions (Carhart-Harris et al., 2016; Barrett et al., 2017). Different serotonergic receptors are expressed in the aRSC including the 5-HT1A, 5-HT2A and 5-HT2C (De Filippo and Schmitz, 2024) and in recent years they have been associated with the modulation of different cognitive processes. Interestingly, it was shown that psychedelic drugs increased theta waves and behavioral flexibility in a 5-HT2A receptors (5-HT2ARs) dependent manner (Pompeiano et al., 1994; Rogers et al., 2025; White et al., 2025) pointing to 5-HT2AR as an important factor in cognitive processes.

5-HT2A receptors modulate memory processes (de Quervain et al., 2003; Zhang and Stackman, 2015). Particularly we have found that 5-HT2ARs are involved in the control of interference during retrieval of episodic “like” memories in the mPFC. In parallel, we have recently shown that aRSC is involved in recognition memory processing (de Landeta et al., 2020). Since 5-HT2ARs are expressed in aRSC, we decided to investigate their role in the aRSC during different phases of a recognition memory task in rats. We found that selective blockade of 5-HT2ARs is required during acquisition for early memory formation, consolidation, and retrieval of long-term memory in the object recognition (OR) task. However, it does not affect the expression of short-term memory. These results suggest that 5-HT2ARs in the aRSC are required for OR memory processing and may represent a potential therapeutic target.

## Methods

### Subjects

Experiments were conducted using male Wistar rats (Universidad de Buenos Aires, Argentina) weighing between 220 and 250 g. Animals were group-housed (three per cage) under controlled environmental conditions (23 °C), with unrestricted access to food and water. A 12-h light/dark cycle was maintained, with lights turned on at 07:00 a.m. Independent groups of animals were used for each time point analyzed. All procedures followed the guidelines established by the U.S. National Institutes of Health for the Care and Use of Laboratory Animals and received prior approval from the Institutional Committee for the Care and Use of Laboratory Animals (CICUAL) of the University of Buenos Aires.

### Surgical procedures

Anesthesia was induced using a combination of ketamine (80 mg/kg) and xylazine (10 mg/kg), administered intraperitoneally. Animals were placed in a stereotaxic apparatus, and the skull was exposed and leveled (lambda and bregma aligned). Bilateral implantation of 22-gauge stainless-steel guide cannulae was performed targeting the aRSC using the following stereotaxic coordinates relative to bregma: AP −3.9 mm, ML ± 0.5 mm, DV −1.8 mm, according to Paxinos and Watson (2007). Cannulae were secured with dental acrylic and sealed with modified 30-gauge metal obturators. Postoperative care included administration of meloxicam (0.2 mg/kg, s.c.) for analgesia and gentamicin (2 mg/kg, s.c.) as an antibiotic. A recovery period of 5–7 days was allowed before behavioral procedures began.

### Drug administration

Infusions were carried out using an internal cannula that extended 1 mm beyond the tip of the guide cannula, connected to 10 μL Hamilton syringes. A total volume of 1 μL per hemisphere was infused at a constant rate of 1 μL/min. The internal cannula was left in place for an additional minute to facilitate diffusion and minimize backflow. MDL 11,939 (MDL, 300 ng/μL) and Ketanserin (Ket, 5 ug/ul) were purchased from Tocris and dissolved in a vehicle (Veh) solution consisting of 5% DMSO in sterile saline and administered bilaterally into the aRSC. Control animals received the same volume of vehicle solution, 5% DMSO in sterile saline solution. The dosages were selected based on preliminary studies and previous publications (Bekinschtein et al., 2013; Kurita et al., 2023, respectively).

### Verification of cannula placement

**FIGURE 1:**
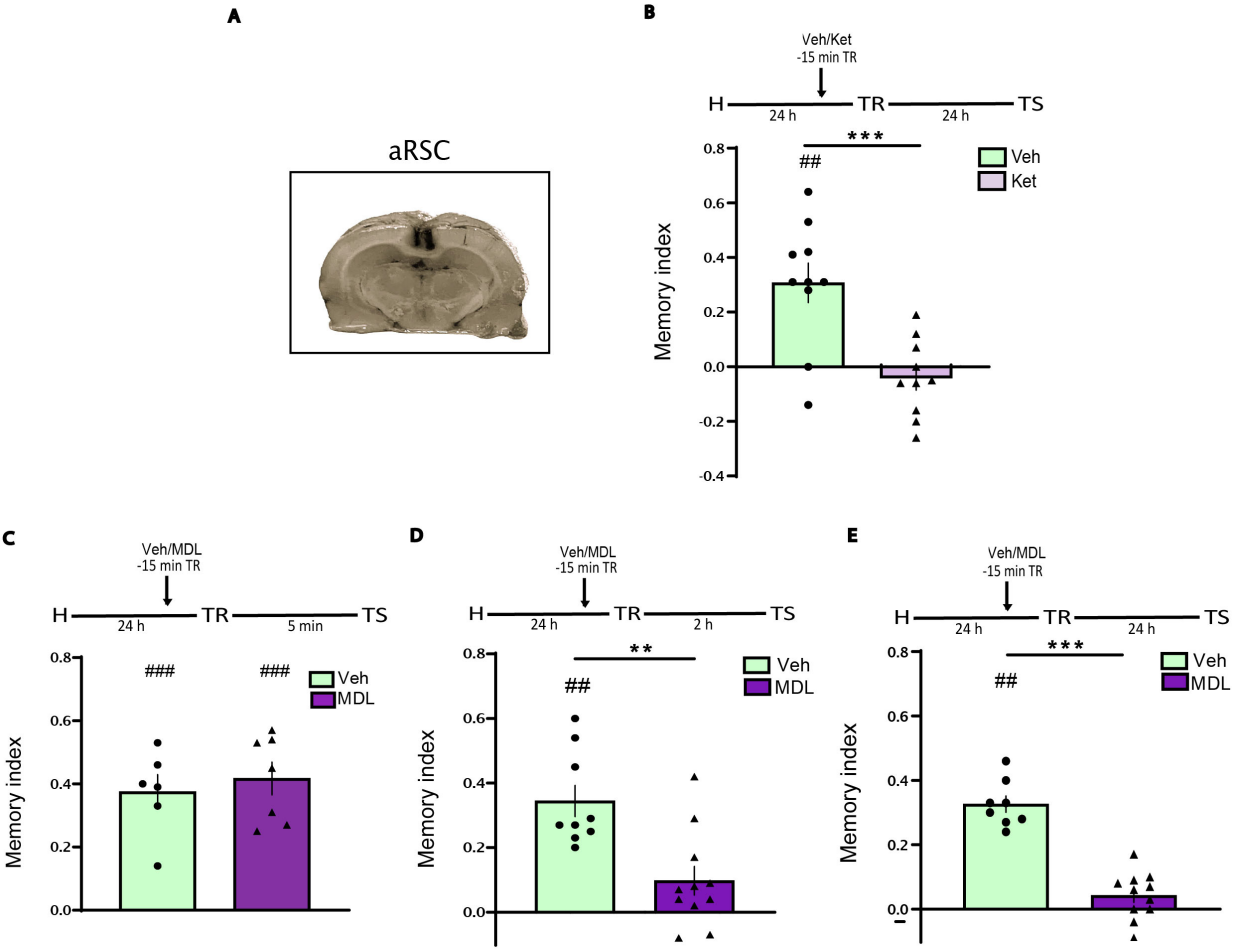
Blocking 5 HT2AR in the aRSC before the training (TR) impairs short and long term recognition memory, but not acquisition. **(A)** Representation of the infusion area. Pictures show the methylene blue infusions area (black) for aRSC. Animals were infused into aRSC 15 min before TR. **(B)** With Ketanserine (Ket) or Vehicle (Veh) and tested 24 h after TR, *n* = 10. **(C)** With MDL11,939 (MDL) or Veh and tested 5 min after TR, *n* = 6–7. **(D)** With MDL or Veh and tested 2 h after TR, *n* = 9–11. **(E)** With MDL or Veh and tested 24 h after TR, *n* = 8–11. Data are expressed as mean ± SEM of the discrimination index. Two tailed Student’s *t*-test: **p < 0.01, ***p < 0.001 vs. Veh; ##*p* < 0.01, ###*p* < 0.001 vs. 0.

At the end of behavioral procedures, cannula placement was verified by infusion of 1 μL of 4% methylene blue dye in saline. Brains were then examined histologically to confirm targeting accuracy. Only data from animals with confirmed bilateral cannula placements within the aRSC were included in the final analyses (see Figure 1A).

### Y-maze object recognition task

Object recognition was assessed using a Y-shaped acrylic maze. Each arm measured 27 cm in length and 10 cm in width, with opaque white walls 40 cm high to prevent access to external spatial cues. The terminal portions of the arms were shortened to 8.5 cm using guillotine doors. Duplicate copies of objects made from plastic and glass were used. Objects were thoroughly cleaned between sessions and randomly assigned to the different phases of the experiments. The heights of the objects ranged from 15 to 25 cm and they varied with respect to their visual and tactile qualities. During the habituation phase, animals were allowed to freely explore the empty maze for 10 min. In the training session, each rat was placed in the start arm and allowed to explore two identical objects placed at the ends of the other two arms for 5 min. The test session, conducted after a retention interval (5 min, 2 h or 24 h), involved a 3-min exploration of two objects: one familiar and one novel. Index was calculated as follow:


$$\text{Index}=\frac{\mathrm{Time}\,\mathrm{exploring}\,\mathrm{Novel}\,\mathrm{Object}-\mathrm{Time}\,\mathrm{exploring}\,\mathrm{Familiar}\,\mathrm{Object}}{\mathrm{Total}\,\mathrm{Object}\,\mathrm{exploration}\,\mathrm{time}}$$

#### Total object exploration time

Only animals that explored each object for at least 15 s during the training session, without showing a preference for any of the objects (<65% of preference for a single object) and that explored for more than 15 s during the test session were included in the analysis. Total exploration times for each experiment and manipulation are shown in Table 1.

**TABLE 1:** Total training and test sessions’ exploration times for each manipulation.

Figure	Group	Training		Test		dF
		Expl time (s)	*P*-value	Expl time (s)	*P*-value	
1A			0.76		0.89	18
	Veh	46.20 ± 13.94		35.30 ± 10.78		
	Ket	48.10 ± 13.79		35.90 ± 10.09		
1B			0.08		0.35	12
	Veh	38.33 ± 14.90		31.50 ± 19.12		
	MDL	49.75 ± 7.55		24.13 ± 9.10		
1C			0.85		0.90	7
	Veh	41.50 ± 18.14		17.00 ± 5.83		
	MDL	39.80 ± 8.52		16.40 ± 7.73		
1D			0.55		0.51	17
	Veh	51.38 ± 12.93		22.25 ± 9.37		
	MDL	56.27 ± 19.60		19.64 ± 7.56		
2A			0.82		0.51	13
	Veh	92.13 ± 17.92		32.88 ± 16.27		
	MDL	89.71 ± 22.33		27.57 ± 13.73		
2B			0.05		0.76	11
	Veh	57.14 ± 9.51		22.86 ± 7.53		
	MDL	67.50 ± 7.50		21.67 ± 5.98		
2C			0.50		0.75	10
	Veh	49.33 ± 14.15		30.17 ± 7.73		
	MDL	42.33 ± 20.30		28.50 ± 9.69		
2D			0.18		0.58	13
	Veh	60.25 ± 22.48		27.00 ± 6.92		
	MDL	78.71 ± 28.39		29.57 ± 10.66		
3A			0.27		0.84	22
	Veh	67.73 ± 24.34		38.82 ± 12.24		
	MDL	56.62 ± 24.10		37.62 ± 15.65		
3B			0.09		0.95	16
	Veh	56.00 ± 23.01		28.13 ± 7.88		
	MDL	42.00 ± 10.45		28.40 ± 10.42		

Mean ± SD exploration time for each experiment during training and test sessions. Results of two-tailed Student’s *t*-test for the exploration time in each experiment.

### Data analysis

Statistical analyses were conducted using unpaired Student’s *t*-test between groups or the theoretical value 0. We used Graph Pad Prism 8 (Graph-pad, USA). Data are reported as mean ± standard error of the mean (SEM), with each data point representing an individual subject. Potential outliers were identified using Grubbs’ test (α = 0.05; GraphPad Software). Only animals with confirmed bilateral cannula placements were included in the final dataset. Each experiment was replicated independently, and reproducibility was confirmed across repetitions. A significance threshold of *p* < 0.05 was applied in all cases.

## Results

### Role 5-HT2A receptors of the aRSC during object recognition memory acquisition

To evaluate the role of aRSC 5-HT2 receptors in object recognition memory, we bilaterally infused Ket (5 μg/μL), a selective 5-HT2 antagonist, into aRSC 15 min before Y-OR training. We found memory impairment at a 24 h test (Figure 1B, Student’s *t*-test. Ket vs. Veh: *p* = 0.0007, *t* = 4.063, df = 18. Ket vs. 0: *p* = 0.3850, *t* = 0.9130, df = 9. Veh vs. 0: *p* = 0.0023, *t* = 4.209, df = 9. n_*Ket*_ = 10, n_*Veh*_ = 10), suggesting that 5HT2 receptors are required during learning for long term memory expression.

In order to determine the specific contribution of 5-HT2A receptor during memory acquisition, we bilaterally infused MDL, a selective 5-HT2A antagonist, into the aRSC 15 min before the training session. Memory performance assessed immediately after training (5 min post-TR) was unaffected, indicating intact immediate memory expression (Figure 1C, Student’s *t*-test. MDL vs. Vehicle: *p* = 0.5880, *t* = 0.5580, df = 11. MDL vs. 0: *p* = 0.0002, *t* = 8.030, df = 6. Veh vs. 0: *p* = 0.0010, *t* = 6.871, df = 5. n_*MDL*_ = 7, n_*Veh*_ = 6). However, when memory was tested at short- (2 h) or long-term (24 h) intervals, a significant impairment was observed (Figures 1D, E, respectively; Figure 1D, Student’s *t*-test. MDL vs. Vehicle: *p* = 0.0015, *t* = 3,730, df = 18. MDL vs. 0: *p* = 0.0545, *t* = 2.177, df = 10. Veh vs. 0: *p* = 0.0001, *t* = 7.055, df = 8. n_*MDL*_ = 11, n_*Veh*_ = 9. Figure 1E, Student’s *t*-test. MDL vs. Vehicle: *p* = < 0.0001, *t* = 8.423, df = 17. MDL vs. 0: *p* = 0.0788, *t* = 1.957, df = 10. Veh vs. 0: *p* = < 0.0001, *t* = 12.73, df = 7. n_*MDL*_ = 11, n_*Veh*_ = 8). These findings suggest that 5-HT2A receptors in the aRSC are not essential for memory acquisition, but are required for its short- and long-term storage.

### Role 5-HT2A receptors of the aRSC during object recognition memory consolidation

**FIGURE 2:**
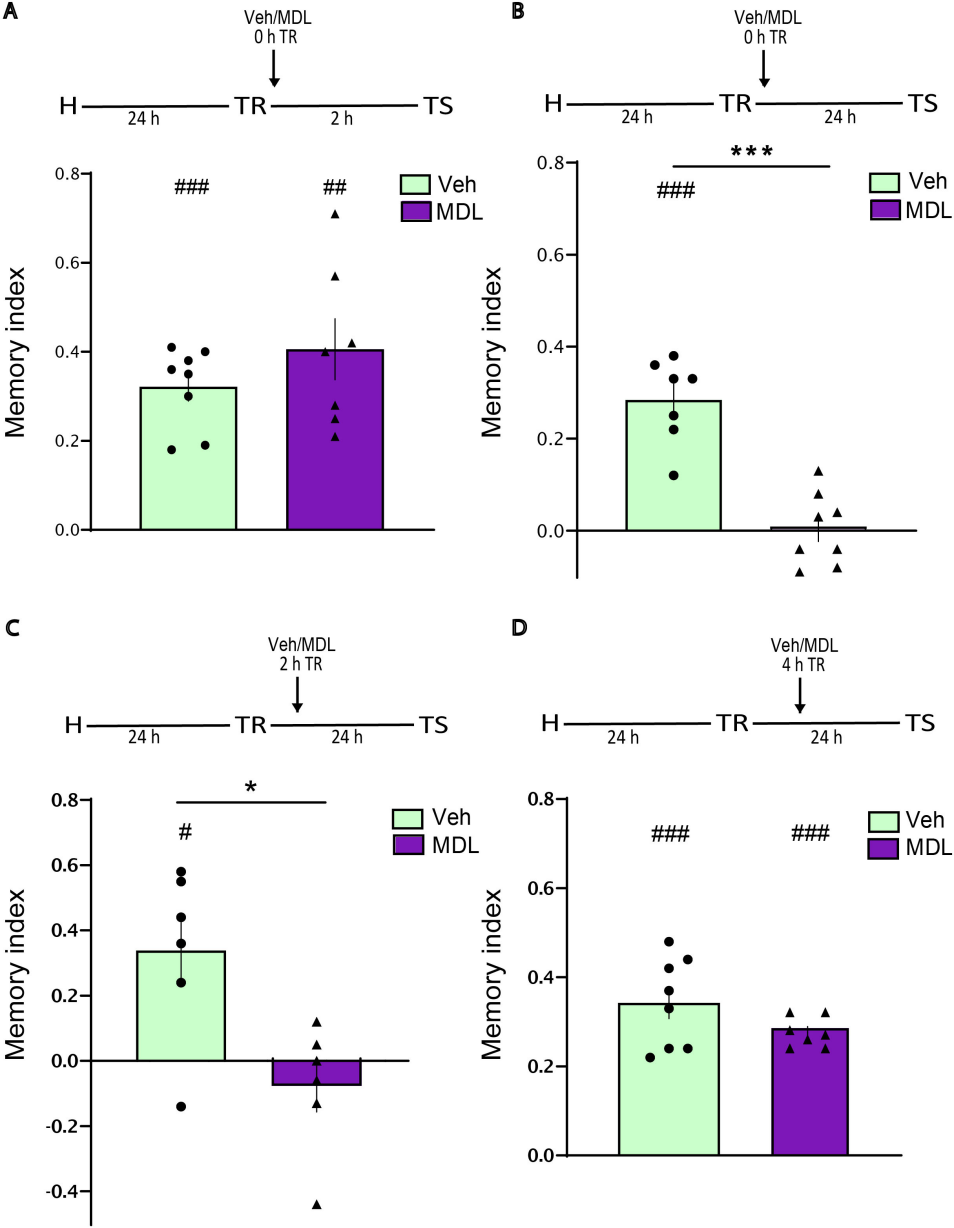
Blocking 5-HT2AR in the aRSC early after TR impairs long-term memory consolidation. Animals were infused with MDL or Veh into aRSC **(A)** immediately after TR and tested 2 h later, *n* = 8–7. **(B)** Immediately after TR and tested 24 h later, *n* = 7–8. **(C)** 2 h after TR and tested 24 h later, *n* = 6. **(D)** 4 h after TR and tested 2 h later, *n* = 8–7. Data are expressed as mean ± SEM of the discrimination index. Two tailed Student’s *t*-test: *p < 0.05, ***p < 0.001 vs. Veh; #*p* < 0.05, ##*p* < 0.01, ###*p* < 0.001 vs. 0.

Next we studied whether the inactivation of aRSC 5-HT2ARs, induced long term Y-OR memory impairments by interfering with the consolidation processes. Thus, we infused MDL at different time points after training. Immediate postTR infusion of MDL into aRSC did not affect short-term memory evaluated at 2 h (Figure 2A, Student’s *t*-test. MDL vs. Veh: *p* = 0.2654, *t* = 1.164, df = 13. MDL vs. 0: *p* = 0.0010, *t* = 5.906, df = 6. Veh vs. 0: *p* = < 0.0001, *t* = 10.03, df = 7. n_*MDL*_ = 7, n_*Veh*_ = 8). However, the same treatment led to a significant impairment in long-term memory 24 h after TR (Figure 2B, Student’s *t*-test. MDL vs. Veh: *p* = < 0.0001, *t* = 6.349, df = 13. MDL vs. 0: *p* = 0.8966, *t* = 0.1347, df = 7 Veh vs. 0: *p* = 0.0002, *t* = 8.129, df = 6. n_*MDL*_ = 8, n_*Veh*_ = 7). A similar result was obtained when MDL was infused 2 h post-training and the retention tested 24 h later (Figure 2C, Student’s *t*-test. MDL vs. Veh: *p* = 0.0118, *t* = 3.070, df = 10. MDL vs. 0: *p* = 0.3861, *t* = 0.9491, df = 5. Veh vs. 0: *p* = 0.0262, *t* = 3.121, df = 5. n_*MDL*_ = 6, n_*Veh*_ = 6). In contrast, infusion of MDL 4 h after TR had no effect on memory performance (Figure 2D, Student’s *t*-test. MDL vs. Veh: *p* = 0.1805, *t* = 1.415, df = 13. MDL vs. 0: *p* = < 0.0001, *t* = 22.50, df = 6. Veh vs. 0: *p* = < 0.0001, *t* = 9.595, df = 7. n_*MDL*_ = 7, n_*Veh*_ = 8). These results suggest the existence of a critical temporal window during which 5-HT2A receptor activity in the aRSC is necessary for memory consolidation.

### Role 5-HT2A receptors of the aRSC during object recognition memory expression

**FIGURE 3:**
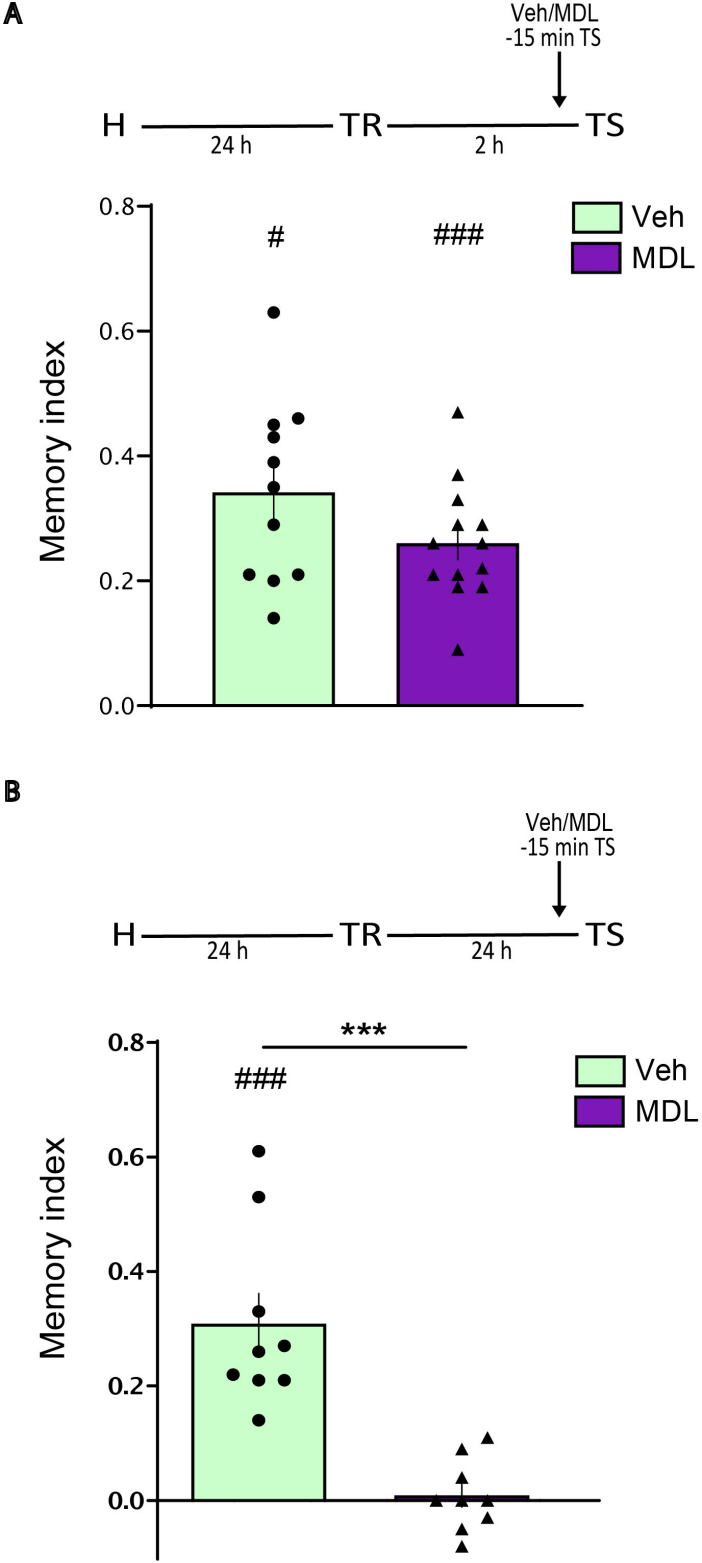
5-HT2A receptors (5-HT2AR) of the aRSC are required during long-term OR memory expression. Animals were infused with MDL or Veh into aRSC 15 min before the test at panel **(A)** 2 h after TR, *n* = 11–13 or **(B)** 24 h after TR, *n* = 9. Data are expressed as mean ± SEM of the discrimination index. Two tailed Student’s *t*-test: ***p < 0.001; #*p* < 0.05, ###*p* < 0.001 vs. 0.

Finally, we also investigated the involvement of aRSC 5-HT2ARs in memory retrieval. We infused MDL into the aRSC 15 min before the test. This manipulation did not affect object recognition memory expression at 2 h (Figure 3A, Student’s *t*-test. MDL vs. Veh: *p* = 0.1148, *t* = 1.642, df = 22. MDL vs. 0: *p* = < 0.0001, *t* = 9.872, df = 12. Veh vs. 0: *p* = < 0.0001, *t* = 7.693, df = 10. n_MDL_ = 13, n_Veh_ = 11), but significantly impaired it at 24 h post-training (Figure 3B, Student’s *t*-test. MDL vs. Veh: *p* = < 0.0001, *t* = 5.299, df = 16. MDL vs. 0: *p* = 0.6791, *t* = 0.4292, df = 8. Veh vs. 0: *p* = 0.0004, *t* = 5.862, df = 8. n_MDL_ = 9, n_Veh_ = 9). These results indicate that 5-HT2A receptors in the aRSC are necessary for the expression of long-term, but not short-term, object recognition memory.

## Discussion

In the present study, we provide novel evidence that 5-HT2ARs within the aRSC play a critical role in object recognition memory. Using the Y-OR task combined with localized administration in the aRSC of Ketanserin, a selective 5-HT2R antagonist, we find that these receptors are required for memory formation. Furthermore, by infusing the selective 5-HT2AR antagonist MDL into the aRSC, we show that these receptors contribute differentially to distinct memory phases, including acquisition, consolidation, and retrieval.

We have previously shown that the aRSC is required for Y-OR memory (de Landeta et al., 2020); however, the molecular mechanisms underlying this process remain largely unknown. Similar to our previous findings, which showed no effect on memory acquisition following aRSC inactivation prior to Y-OR training, we found that 5-HT2AR signaling is not required for acquisition *per se*, since when animals were tested immediately after the training session, they solved the task independently of the pharmacological treatment. Interestingly, however, when we evaluated memory at a 2 and 24 h retention period, we found that 5-HT2ARs blockade prior to the training session impaired the resolution of the task. These results suggest that 5-HT2AR signaling in aRSC might participate in mechanisms underlying the more stable forms of memory. The mechanisms through which 5-HT2ARs regulate memory stability appear to depend on the temporal window of receptor activation. Our findings indicate that 5-HT2AR activity is required in a time dependent manner. This observation suggests that post-acquisition 5-HT2AR signaling contributes primarily to the consolidation of memory rather than to the formation of short-term memory. Furthermore, 5-HT2AR signaling appears to be selectively involved in the retrieval of long-term, but not short-term, memory.

Our understanding of 5-HT2ARs’ function in memory processes is still very limited and quite complex. In mice it was shown that post-training activation of 5-HT2ARs in the hippocampus enhanced consolidation of object memory, though pre-test activation does not affect retrieval of object memory, yet delayed retrieval of spatial memory (Zhang and Stackman, 2015; Stackman et al., 2016). In rats, previous studies have demonstrated that blocking 5-HT2AR in the mPFC before testing does not impair spontaneous novel object recognition, but it does impair recency discrimination and object in context during memory retrieval (Bekinschtein et al., 2013). While the mPFC provides top-down control to learn associations that are pivotal to the selection of relevant memory to guide decision-making processes, the aRSC, has a more direct role in memory processes due to its extensive connectivity with the hippocampus and similarly to the mPFC it is suggested that it plays a role in the integration of information during memory acquisition.

Though mPFC appears to participate in recognition memory when associations among different aspects of the event are relevant, its activity and modulation by 5-HT are not necessary for object recognition retrieval *per se* (Bekinschtein et al., 2013; Cross et al., 2013). However, this is not the case for aRSC- 5-HT2AR. Consistently with prior studies that have shown that aRSC activity is necessary for the consolidation and expression of “what” information (de Landeta et al., 2020) aRSC-5-HT2ARs participate in the more basic functions of recognition memory. These results are consistent with previous literature that indicates a role of aRSC in recognition memory beyond spatial information. Since recognition memory, which is part of episodic memory, is affected in cognitive decline associated with aging and neurodegenerative disorders like Alzheimer’s disease, identifying one of the main serotoninergic receptors as a key modulator of aRSC function highlights the serotoninergic system as a potential therapeutic target.

## Data Availability

The raw data supporting the conclusions of this article will be made available by the authors, without undue reservation.
